# On the Environmentally Friendly Synthesis of 2-Hydroxyethyl Furan-5-Carboxylic Acid (MHEF) and bis(2-Hydroxyethyl) Furan-2,5-Dicarboxylate (BHEF)

**DOI:** 10.1002/open.202400507

**Published:** 2025-01-28

**Authors:** Francesco Raboni, Andrea Oliveri, Valeria Marisa Rocca, Lisa Moni, Virender Kumar, Cristiano Varrone, Alessandro Pellis

**Affiliations:** [a] Dipartimento di Chimica e Chimica Industriale Università degli Studi di Genova Via Dodecaneso 31, 16146, Genova, Italy; [b] Department of Chemistry and Bioscience Aalborg Universitet Fredrik Bajers Vej 7H, 9220 Aalborg Øst, Denmark

**Keywords:** 2-hydroxyethyl furan-5-carboxylic acid (MHEF), bis(2-hydroxyethyl) furan-2,5-dicarboxylate (BHEF)

## Abstract

To better understand how the biocatalyzed depolymerization of polyesters works, model molecules are needed to develop activity assays and determine enzymatic kinetic parameters. In this communication the chemical synthesis and characterization of 2-hydroxyethyl furan-5-carboxylic acid and bis(2-hydroxyethyl) furan-2,5-dicarboxylates as potential model molecules to further study the enzymatic depolymerization of poly(ethylene furanoate) was investigated.

## Graphical abstract

**Figure:**
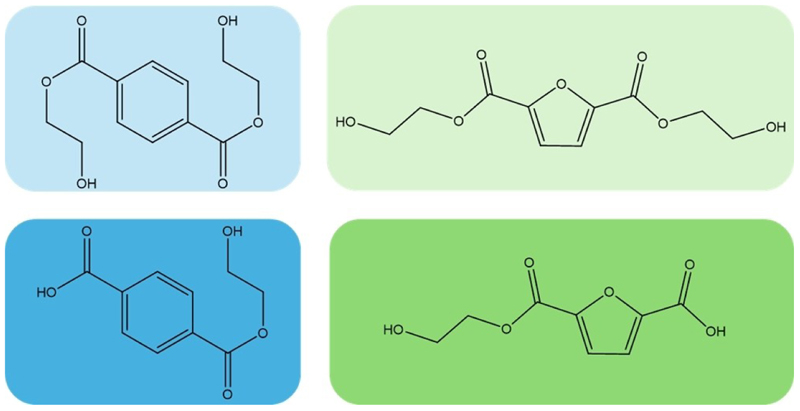
To better understand how the biocatalyzed depolymerization of polyesters works, model molecules are needed to develop activity assays and determine enzymatic kinetic parameters. In this communication the chemical synthesis and characterization of 2-hydroxyethyl furan-5-carboxylic acid and bis(2-hydroxyethyl) furan-2,5-dicarboxylates as potential model molecules to further study the enzymatic depolymerization of poly(ethylene furanoate) was investigated.

Currently 99% of all plastic is produced from fossil fuel.^[1]^ Although amounting for less than 10%^[2]^ of global oil consumption, within the last 70 years plastic production increased more than 200-fold.^[3]^ For these reasons nowadays, due to the increasing concerns regarding global warming and depletion of fossils resources, there is a growing need for biobased and renewable plastics, as well as efficient methods to recover and recycle the monomers. Bioplastics are classified as either Biobased, Biodegradable or both.^[1]^ In a biobased plastic, carbon and hydrogen atoms derive from biomasses and vegetal compounds, mainly carbohydrates and lignin. These molecules are then processed to obtain monomers suitable for polymerization such as furan dicarboxylic acid (FDCA), used in the synthesis of poly(ethylene furanoate) (PEF), a biobased alternative to the fossil derived poly(ethylene terephthalate) (PET), one the most produced plastics for bottles and packaging. PEF is currently one of the most promising and studied biobased polyesters, it has shown superior properties^[4]^ compared to PET and life cycle assessment was performed showing a reduction between 30–50% of greenhouse gas emission and energy use.^[5]^ Moreover, although terephthalic acid (TPA) could technically be produced from biomasses, currently it’s still hard to convert lignocellulose moieties into this monomer,^[6]^ rendering its production economically unsustainable.^[7]^ For this reason, there is an increasing interest in developing and engineering enzymes useful to depolymerize these materials to their constituent monomers. To do so it is necessary to carefully study the molecular mechanism and kinetic of these enzymatic processes and for this reason high purity model compounds like the ones presented in this work are needed to investigate the molecular mechanisms at the basis of the biocatalyzed depolymerization reaction. For example, the enzymatic depolymerization of PEF has already been reported,^[8,9]^ however, the mass balance, effect of the oligomers on the overall hydrolysis and kinetics could not be ascertained due to the non-availability of commercial MHEF and BHEF.

## Results and Discussion

**Figure 1:**
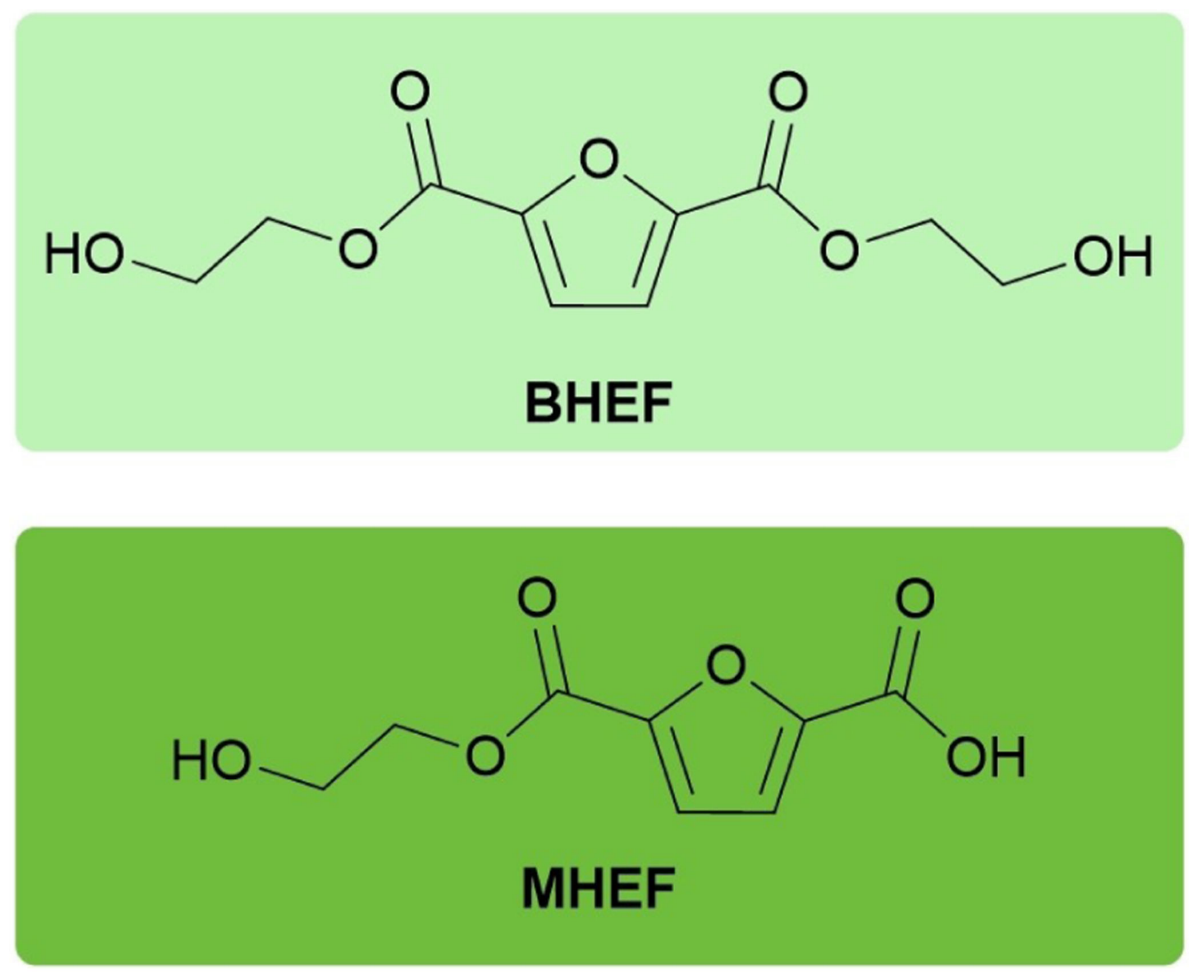
Structures of the synthesized compounds bis(2-hydroxyethyl) furan-2,5-dicarboxylate (BHEF) (top, light green) and 2-hydroxyethyl furan-5-carboxylic acid (MHEF) (bottom, dark green).

Aiming to synthesize mono and diesters of FDCA and ethylene glycol (EG) (Figure 1), the constituent monomers of PEF, the information regarding the synthesis of these molecules available in the literature is sparse, especially regarding the preparation of mono esters.

Therefore, the preparation of bis(2-hydroxyethyl) terephthalate (BHET) was selected as the starting point. BHET can be synthesized with several protocols however, most reactions dealing with BHET do not report yields, are under patent and were found to be far from the ideal of eco-sustainability since they either use harmful reagents, harsh conditions, expansive metal catalysts or using various derivatives and protective groups.

Moreover, some of these methodologies are directed towards a large-scale industrial synthesis and use harmful reagents like ethylene oxide,^[10]^ or obtain this monomer by chemically depolymerizing PET in different conditions^[11–13]^ (high pressure and temperature, and using metallic catalyst). Others reported procedures include transesterification of dimethyl terephthalate either using metal catalysis^[14]^ or imidazolium salts,^[15]^ and the alkoxycarbonylation of aryl iodides with Mo(CO)_6_.^[16]^ The direct rection between TPA and EG was only performed in a flow reactor with a yield of 97.5% at 180 °C in 9 h.^[17]^ The furane counterpart was again obtained either from the metal-catalyzed transesterification of the corresponding dimethyl ester^[18]^ or from 5-hydroxymethyl-2-furfuraldehyde, using a multi-step approach.^[19]^

The MHET synthesis was less reported than the diester, ethylene oxide^[20]^ was again used but only alkoxycarbonylation^[16]^ and protective groups^[21]^ were found to be an effective strategy to selectively obtain the desired product; no procedure for the obtainment of MHEF was instead found.

**Scheme 1:**
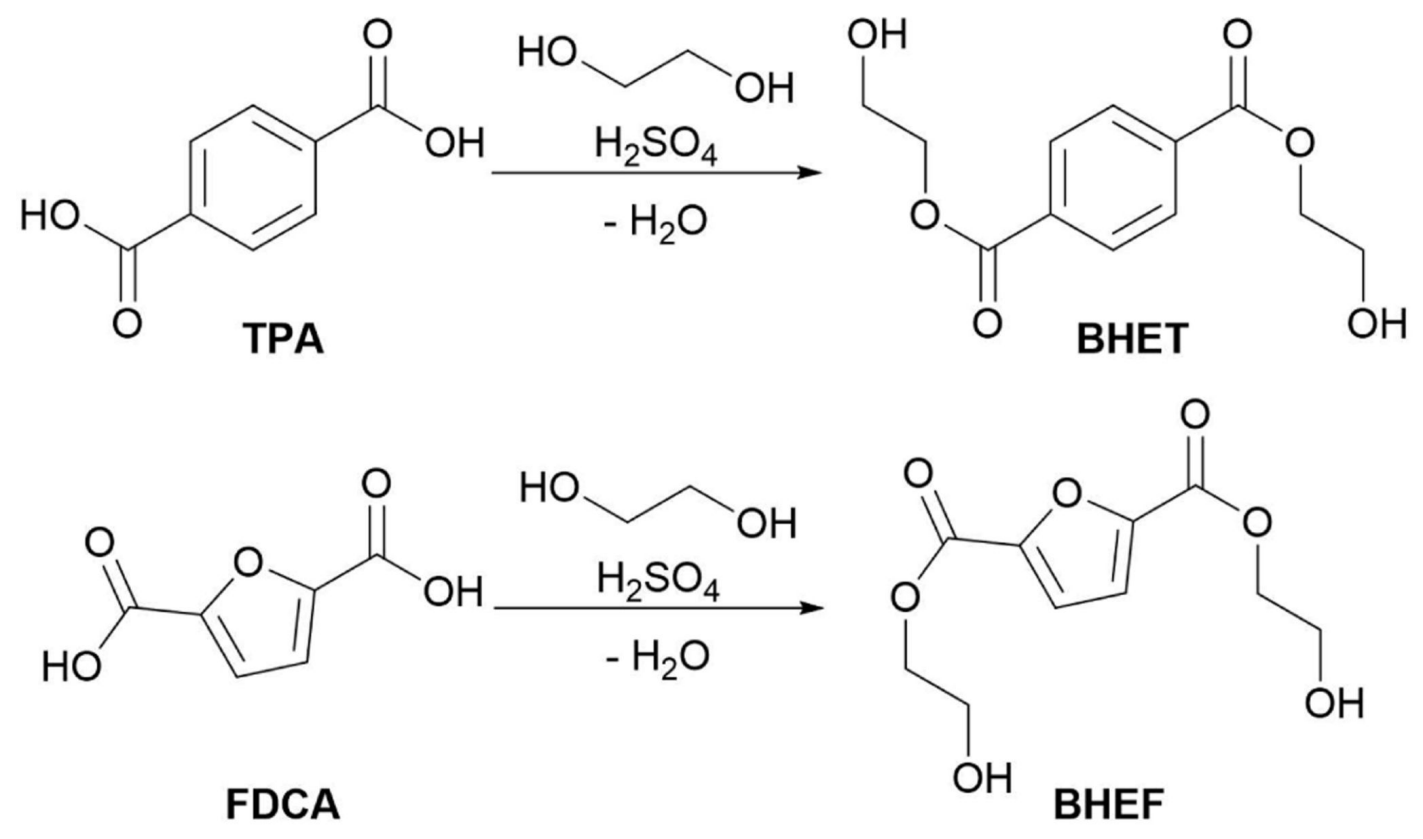
Synthesis of bis hydroxyethyl diesters through Fischer esterification.

This led to the search for simpler and straightforward strategies to obtain these molecules. For this reason, following the green chemistry principles, a simple acid-catalyzed Fischer esterification (Scheme 1) was carried out.

Once the solution cools down, NaHCO_3_ is added to neutralize and deprotonate any residual diacid with the diester that is then extracted with AcOEt. This reaction in literature was carried out only on FDCA using benzene as the reaction solvent, with a total yield of 54%.^[22]^ When carrying out the reaction on TPA using toluene as a safer alternative to benzene (Table 1, entry 1) no conversion was observed as the TPA was unable to dissolve in the solvent and the small amount of EG. Seeing that the reaction in solvent was not working, toluene was removed from the reaction system and a 10-fold excess of EG was used (Table 1, entry 2). Using this protocol after ~30 min the reaction mixture became homogeneous and, after 24 h the product was recovered with 80% yield. The limitation of this methodology lies in the fact that EG partially dissolves in ethyl acetate, carrying on with the organic phases, so to completely remove it from the product a lyophilization step is required (white solid, purity > 95%). The reaction was then further optimized (Table 1, entry 3) by reducing the reaction time to 6 h. After the successful optimization of the BHET synthesis, the process was scaled-up by performing the reaction on 1 gram of TPA (Table 1, entries 4 and 5) that led to a yield of 98% and a 95% purity of the recovered compound. The procedure was then applied for the synthesis of BHEF. The first synthesis (Table 1, entry 6) led to a yellow solid (BHET was instead white), although the amount of coloured impurity is negligible and almost not detectable at ^1^H-NMR, the reaction was repeated (Table 1, entry 7) using a 2-necked flask equipped with a nitrogen inlet. The reaction proceeded in the same fashion, providing a crystalline white solid; most likely the atmospheric oxygen is capable of oxidizing and therefore partially degrading the furan ring. Lastly, in Table 1, entry 8 the scale-up of the reaction on 1 gram is reported obtaining analogues results. Using FDCA as the aromatic diacid the extra 8 equivalents of glycol reported on entry 4 were not needed since FDCA is more polar and has a higher solubility than TPA.

**Scheme 2:**
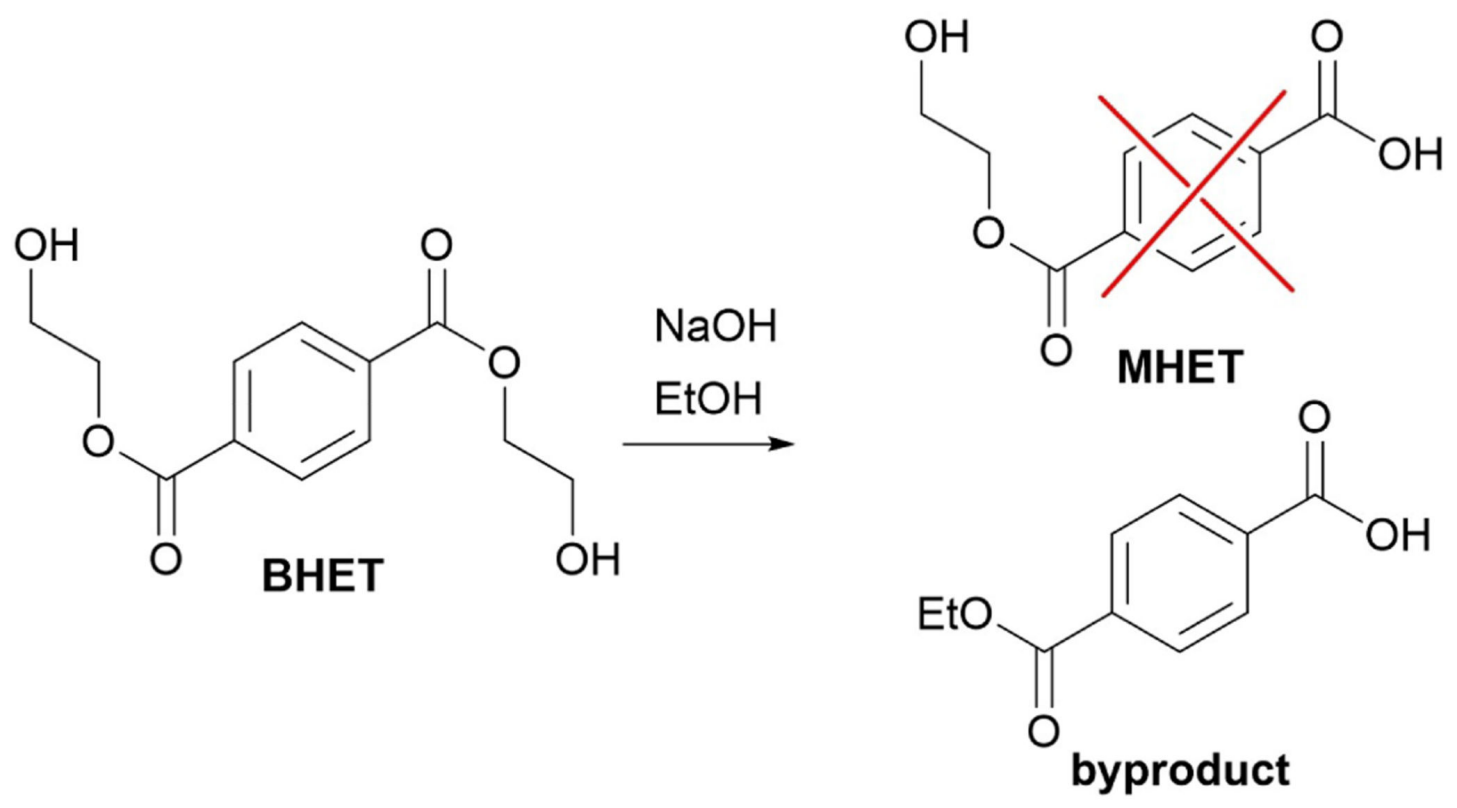
Formation of the hydrolyzed transesterification byproduct.

After the synthesis of BHEF, the synthesis of the monoesters was carried out. This proved to be particularly challenging because, while the two reagents in stoichiometric amounts should generate only the desired product, there is always a certain percentage of diester and residual diacid. The latter is impossible to extract separately and extremely difficult to remove using column chromatography because of its similarity and interactions with the product. While for 2-hydroxyethyl furan-5-carboxylic acid (MHEF) there were no reported synthesis, for 2-hydroxyethyl terephthalic acid (MHET) there are few synthetic routes available. Again, most of these strategies were under patent, did not report any yield and used ethylene oxide or metal catalysts. Several synthetic strategies reported in Table 2 were attempted. Fischer esterification was not possible this time due to the need to keep the molar ratio close to 1 and lack of a suitable solvent. Therefore, we tried the opposite route, a basic hydrolysis of the previously synthesized diester. In entries 1–5 different hydrolytic conditions, changing solvent, base, time, and temperature were tested, but sadly none of these were able to provide an acceptable yield with high purity and low diacid content. Only entry 3 produced a good yield with excellent purity however, the recovered product was not the hydroxyethyl monoester, but the ethyl monoester derived from the side reaction with ethanol (Scheme 2) which was used as solvent. Thus, the base-catalyzed transesterification appears to be much faster than the subsequent hydrolysis reaction.

**Scheme 3:**
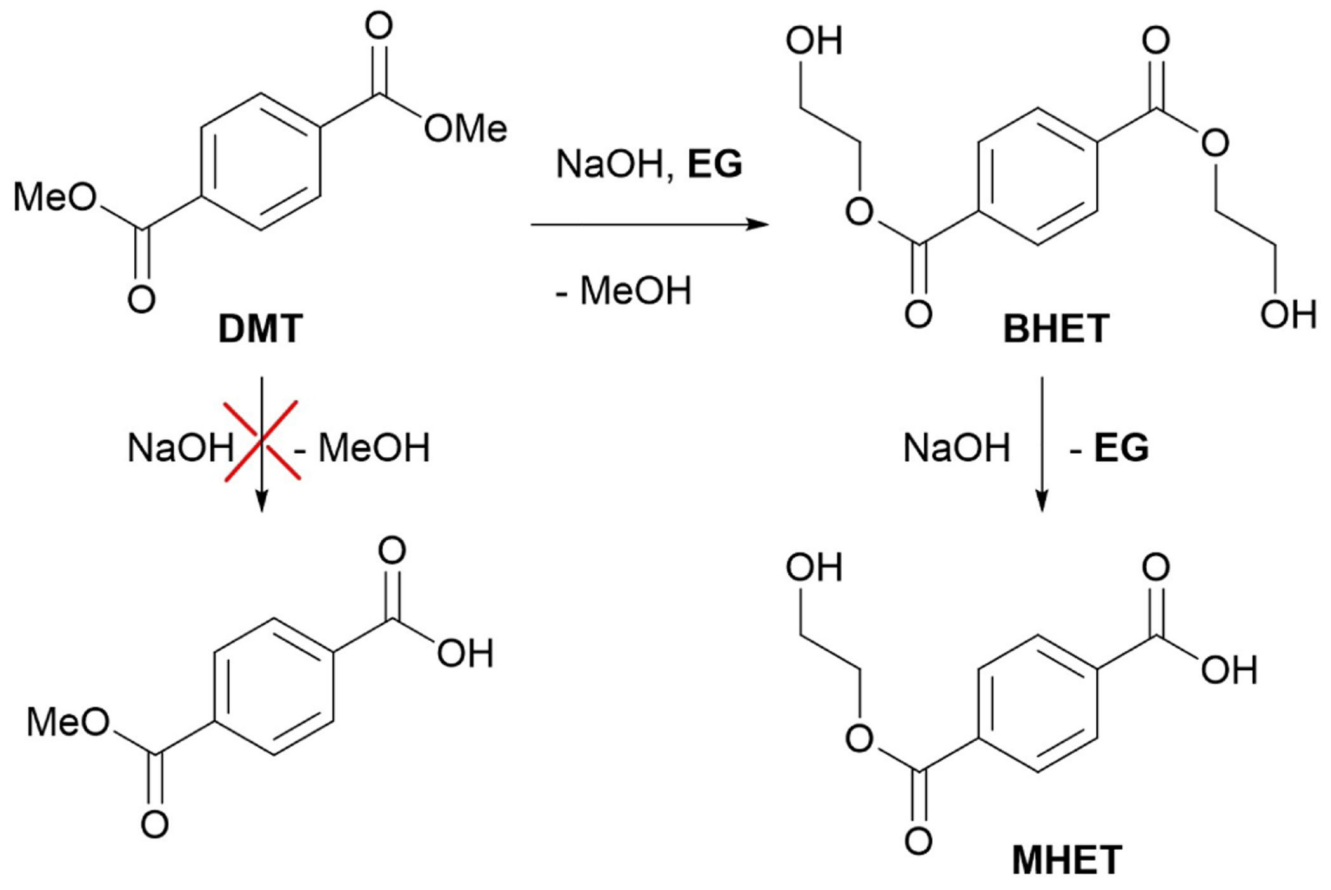
Hydrolysis-followed transesterification to obtain monoesters.

Due to the lack of success with these strategies, three different coupling methods to obtain the desired product were carried out. A sub-stoichiometric amount of TPA to preferably obtain the (easier to remove) diester instead of the diacid was used. In Table 2, entry 6 the results of a Steglich esterification^[23]^ are reported which led to the formation of short oligomers (see ESI, Figure S1) and only traces of the desired product could be recovered. Table 2, entry 7 relates to the activation of the reagents with thionyl chloride however, we recovered only a small fraction from aqueous phases containing mostly TPA since most of the substrate was again converted to a polyester. To avoid this polycondensation, we decided to use 2-iodoethanol in basic conditions to selectively achieve a mono substitution;^[24]^ the reaction should have lasted 4 h but even after 24 h only traces of product were found on TLC. Then, building on the previous observations, the hydrolysis of dimethyl terephthalate (DMT) using EG as solvent (Table 2, entry 9) was performed. Despite the high viscosity and the reaction proceeding mostly in heterogeneous condition, due to the low solubility of DMT, a large amount of monoester (50% yield), contaminated with some TPA, a small amount of residual DMT and mostly BHET was obtained, proving that transesterification proceeds faster than the hydrolysis (Scheme 3).

After the initial extraction with AcOEt to remove the diesters it is possible to eliminate the glycol either by lyophilization or vacuum distillation. Once the glycol is removed MHET is dissolved in basic water, precipitated adding HCl and filtered. Since this molecule is intended to be used in enzymatic assays, purity must be exceptionally high, with inverse phase chromatography and even solid phase extraction (SPE) cartridges can be of great use to remove any remaining TPA, allowing the obtainment of pure MHET (> 99%) with a 50% yield. Moreover, the cartridge can be run multiple times and uses water as solvent. Lastly in Table 2, entry 10, the same methodology to the synthesis of MHEF by starting from dimethyl furan dicarboxylate (DMFu) was applied. In this case the reaction was again completely homogeneous thanks to the higher polarity of the furan ring, sadly this also caused the MHEF to not precipitate in acidic water as opposed to MHET, moreover the small loading SPE were not enough to separate it from residual FDCA. We then resorted to use flash chromatography to separate and purify the product, obtaining an off-white solid with up to 69% yield on a 3-gram scale. To assess the purity, the obtained compounds were characterized by mono- and bidimensional NMR, HPLC-MS, IR and GC-MS when possible. Melting point determination was also performed.

## Conclusions

In this work a facile and environmentally friendly procedure to obtain the glycol-containing diesters BHET and BHEF, with high yield and purity above 95% was reported. Furthermore, a novel mild strategy to obtain monoesters in good yield from symmetric aromatic diesters employing a hydrolysis-followed transesterification reaction. In this second case, the successful purification process has also proven to be particularly complicated because of the poor solubility of the products and the high boiling point of the EG.

## Supplementary Material

ESI

## Figures and Tables

**Table 1:** Fischer esterification of terephthalic acid or furan dicarboxylic acid with ethylene glycol.

Entry	Monomer	Diacid/EG ratio	Time [h]	Yield %	Scale [mg]
Literature^a^	FDCA	1:3	24	54	500
1^b^	TPA	1:3	24	Traces	500
2	TPA	1 :30	24	90	500
3	TPA	1 :30	6	90	500
4	TPA	1 :30	6	65	1000
5	TPA	1 :38	6	98	1000
6	FDCA	1 :30	6	72	500
7^c^	FDCA	1 :30	6	75	500
8^c^	FDCA	1 :30	6	72	1000

H_2_SO_4_ (95%) was used as catalyst, 1–2 drops on less than 1 g scale, 3–5 drops on 1 g scale. Temperature is 110°C.

[a] Solvent used: benzene.

[b] Solvent used: toluene.

[c] Under nitrogen atmosphere

**Table 2:** Strategies to obtain monoesters of terephthalic acid or furan dicarboxylic acid.

Entry	Reagent A	Reagent B	Ratio	Reagent C	Solvent	T [C°]	Time [h]	% Yield of monoester
1	BHET	NaOH	1:1	–	Water	R.T.	18	Traces
2	BHET	NaOH	1:1	–	Glycol	R.T.	18	Traces
3	BHET	NaOH	1:1	–	Ethanol	R.T.	18	50 ^a^
4	BHET	LiOH	1:1	–	Water/acetone	80	2.5	10
5	BHET	LiOH	1:1	–	Water/THF	80	2.5	22
6	TPA	EG	1:1.3 ^b^	DCC	DMF	0 – R.T.	6	Traces
7	TPA	EG	1:1.3 ^b^	SOCl_2_	EG	R.T.	26	0
8	TPA	Iodoethanol	1:1.3	NaHCO_3_	DMF	85	25	Traces
9	DMT	NaOH	1.15:1	–	EG	R.T.	14	50
10	DMFu	NaOH	1.15:1	–	EG	R.T.	14	69

a Not the desired product

b Ratio is Reagent A to Reagent C, EG is in large excess

## Data Availability

The data that support the findings of this study are available in the supplementary material of this article.
