# Supplementary material for: On the Environmentally Friendly Synthesis of 2‐Hydroxyethyl Furan‐5‐Carboxylic Acid (MHEF) and bis(2‐Hydroxyethyl) Furan‐2,5‐Dicarboxylate (BHEF)
Source: ChemistryOpen. 2025 Jan 28;14(8):e202400507. doi: 10.1002/open.202400507 (PMC7617567; doi:10.1002/open.202400507)
Supplement: Supplementary file 1 — Supporting Information [file OPEN-14-e202400507-s001.pdf]

ChemistryOpen

Supporting Information

On the Environmentally Friendly Synthesis of 2-Hydroxyethyl Furan-5-Carboxylic Acid (MHEF) and bis(2-Hydroxyethyl) Furan-2,5-Dicarboxylate (BHEF)

Francesco Raboni, Andrea Oliveri, Valeria Marisa Rocca, Lisa Moni, Virender Kumar, Cristiano Varrone, and Alessandro Pellis*

Electronic Supplementary Information

for

On the environmentally friendly synthesis of 2-hydroxyethyl furan-5-carboxylic acid and bis(2-hydroxyethyl) furan-2,5-dicarboxylate

Francesco Raboni[a], Andrea Oliveri[a], Valeria Marisa Rocca[a], Lisa Moni[a],Virender Kumar[b], Cristiano Varrone[b], Alessandro Pellis[a]*

[a] Università degli Studi di Genova, Dipartimento di Chimica e Chimica Industriale, via Dodecaneso 31, 16146, Genova, Italy.

[b] Aalborg Universitet, Department of Chemistry and Bioscience, Fredrik Bajers Vej 7H, 9220 Aalborg Øst, Denmark.

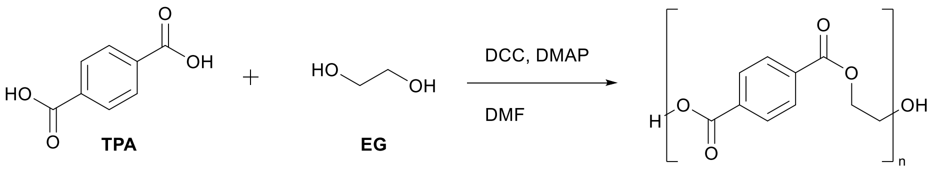

Scheme S1. Formation of oligomers following Steglich esterification.

Materials and methods

Reagents and solvents

Commercial products were used directly without further purifications. TPA (Merck 99%), FDCA (Apollo Scientific 99%), DMT (Merck 99%), DMFu (TCI, >98%) EG (WWR 98%), DCC (Merck 99%), thionyl chloride (Fluka 99%). Toluene (Merck 99%), petroleum ether (Carlo Erba 99%), EtOAc (Merck 99%), DMSO (Merck 99%), MeOH (VWR Chemicals 99%), EtOH (Carlo Erba 99%), DMF (Riedel de Haen 99%), THF (Riedel-de Haen 99%, stabilized with 250 ppm of BHT), acetic acid (Merck 99%), acetone (Merck 99%). $CDCl_3$ (Euristop 99.8% D), DMSO-d6 (Euristop 99.8% D).

Routine analysis

NMR spectra were recorded on a JEOL JNM-ECZ400R (400 MHz for ^{1}H e 100 MHz for ^{13}C) with a Royal HFX probe. $CDCl_3$, DMSO-d6, or D_2O were used as solvents, employing tetramethylesilane (TMS) as the internal standard (0.00 ppm) and the solvent peak for carbon spectra. Chemical shifts (δ) were reported in parts per million (ppm) and coupling constants (J) in Hertz. Peak assignments were also made with the aid of 2D HSQC and HMBC. IR spectra were recorded with a Perkin Elmer Spectrum 65 FT-IR (Fourier Transform Infrared) in ATR (Attenuated Total Reflectance) mode, range 4000-600 cm^{-1}, 16 scans for spectra, resolution: 1 cm^{-1}.
High Performance Liquid Chromatography (HPLC) 1100 Agilent series coupled with an electrospray mass spectrometry Microsaic 4000 MiD Series were used to analyze the synthesized samples.

The analytes were separated using the HPLC equipped with an ACE Excel C18-AR phenylic reverse phase column of 150 × 3 mm and 3µm particle diameter. A gradient elution was used by Mobile phase channel A ddH2O water with 0.1% formic acid (FA), and mobile phase channel B LC/MS grade acetonitrile with 0.1% FA (initial time: A=90% - B=10%, in 20min B= 100%). A VWD Detector was used at 220nm and 241nm. Mass Spectrometry was used with positive ionization to perform a complete scan from 100 to 800 m/z.
GC-MS analyses were performed on a Shimadzu GC-MS QP2010 SE using a HI-5 ms column (0.25 mm, 0.25 mm i.d., and 30 m). Analysis conditions are as follows: solvent delay 2.5 min, mass range 35−600, injector temperature 250 °C, detector temperature 250 °C, MS temperature around 250 °C, starting temperature 150 °C, starting time 3 min, temperature gradient 25 °C min^{-1} , final temperature 300 °C, flux through column 1.0 mL min^{-1} , split ratio 1:10, sample concentration 0.1 mg mL^{-1} , and amount injected 1 mL. TLC analyses were carried out on glass supported silica gel plates (Merck 60 F254 0.25 mm) and viewed at UV (λ = 254 nm), developed with basic potassium permanganate (solution prepared dissolving 1.5 g $KMnO_4$, 10 g K_2CO_3, 1 mL NaOH 10% in 200 mL water). Column chromatography was done using the “flash” methodology using Ge Duran SI 60Å (230–400 mesh).

Methods

Synthesis of *Bis hydroxyethyl terephthalate* (BHET) *a*nd *Bis hydroxyethyl furan-2,5-dicarboxylate* (BHEF)

For the synthesis of BHEF a nitrogen atmosphere is necessary to obtain a white product, a two-neck flask is used with N_2 inlet on the side and a vacuum glass adapter on the main neck to create an outgoing flux of N_2.

A stirring heterogeneous mixture of EG (12 or 16 mL, 180 or 240 mmol) and the corresponding diacid (FDCA or TPA, about 6 mmol, 1 g) are heated at 110 °C under magnetic stirring, 4 drops of H_2SO_4 (95%) are then added and the solution is left stirring for 6 hours. After about 30 minutes all the acid should be dissolved and the reaction homogeneous. Once cool, the reaction is brought to pH 8 with a saturated solution of $NaHCO_3$, diluted with 30 mL of deionized water and then extracted in a separatory funnel 3 times with 30 mL of AcOEt. After solvent evaporation the semi-solid product is dissolved in 60 mL of distilled water and lyophilized to obtain the pure diesters (1.568 g, 71.5% yield, 97% pure BHEF) (1.823 g, 98% yield, 95% pure BHET) as white solids.

Synthesis of *2-hydroxyethyl terephthalic acid* (MHET)

To a stirring mixture of diester DMT (1 g, 5.15 mmol) in the corresponding alcohol EG (20 ml), is added dropwise a solution of NaOH (200 mg, 5 mmol) in EG (5 mL). After 14 hours the reaction is diluted with 50 mL of water, residual NaOH is neutralized with HCl and the pH brought to 8 with saturated $NaHCO_3$, the

solution is then extracted four times with 35 mL of AcOEt to remove residual diesters. To remove excess glycol the solution is brought to pH 7, diluted to 400 mL with deionized water and lyophilized, more cycles could be necessary; alternatively, water and excess glycol can be removed by vacuum distillation (30 mbar, 130 °C). The product is then recovered, after dissolving it in few basic water and inducing precipitation with HCl, by vacuum filtration. If necessary, to remove residual TPA a SPE cartridge (Supelco, Discovery DSC-18) was used with water as solvent. The desired product was obtained as a white solid with a yield of 50%, 539.2 mg and a purity of 99%.

Synthesis of ***2-hydroxyethyl furan-5-carboxylic acid*** (MHEF)

To a stirring mixture of diester DMFu (3 g, 16.3 mmol) in the corresponding alcohol EG (50 mL), is added dropwise a solution of NaOH (600 mg, 15 mmol) in EG (15.5 mL). After 14 h the reaction is diluted with 100 mL of deionized water, residual NaOH is neutralized with HCl and the pH brought to 8 with saturated $NaHCO_3$, the solution is then extracted four times with 75 mL of AcOEt to remove residual diesters. To remove excess glycol the solution is brought to pH 7, diluted to 400 mL with water and lyophilized, more cycles could be necessary; alternatively, water and excess glycol can be removed by vacuum distillation (30 mbar, 130°C). The crude product is then purified by flash chromatography (AcOEt-EtOH 1:1 + 2,5% acetic acid). After solvent evaporation, residual acid is removed by trituration with $CHCl_3$ to give the desired product as a white solid with a yield of 69%, 2.263 g, 96% pure.

Characterization of compounds

NMR spectra

Bis hydroxyethyl terephthalate

MW: 254.24 g/mol

White solid, MP: 105-109°C (Teo 106°C)

^{1}H NMR (400 MHz, DMSO-d_6) δ 8.09 (s, 4H), 4.92 (OH) (t, *J* = 5.7 Hz, 2H), 4.31 – 4.26 (m, 4H), 3.68 (td, *J* = 5.7, 4.5 Hz, 4H).

^{13}C NMR (101 MHz, DMSO-D_6) δ 165.72 (2CO), 134.30 (2Cq), 130.07 (4CH), 67.57 ($2CH_2$), 59.51 ($2CH_2$).

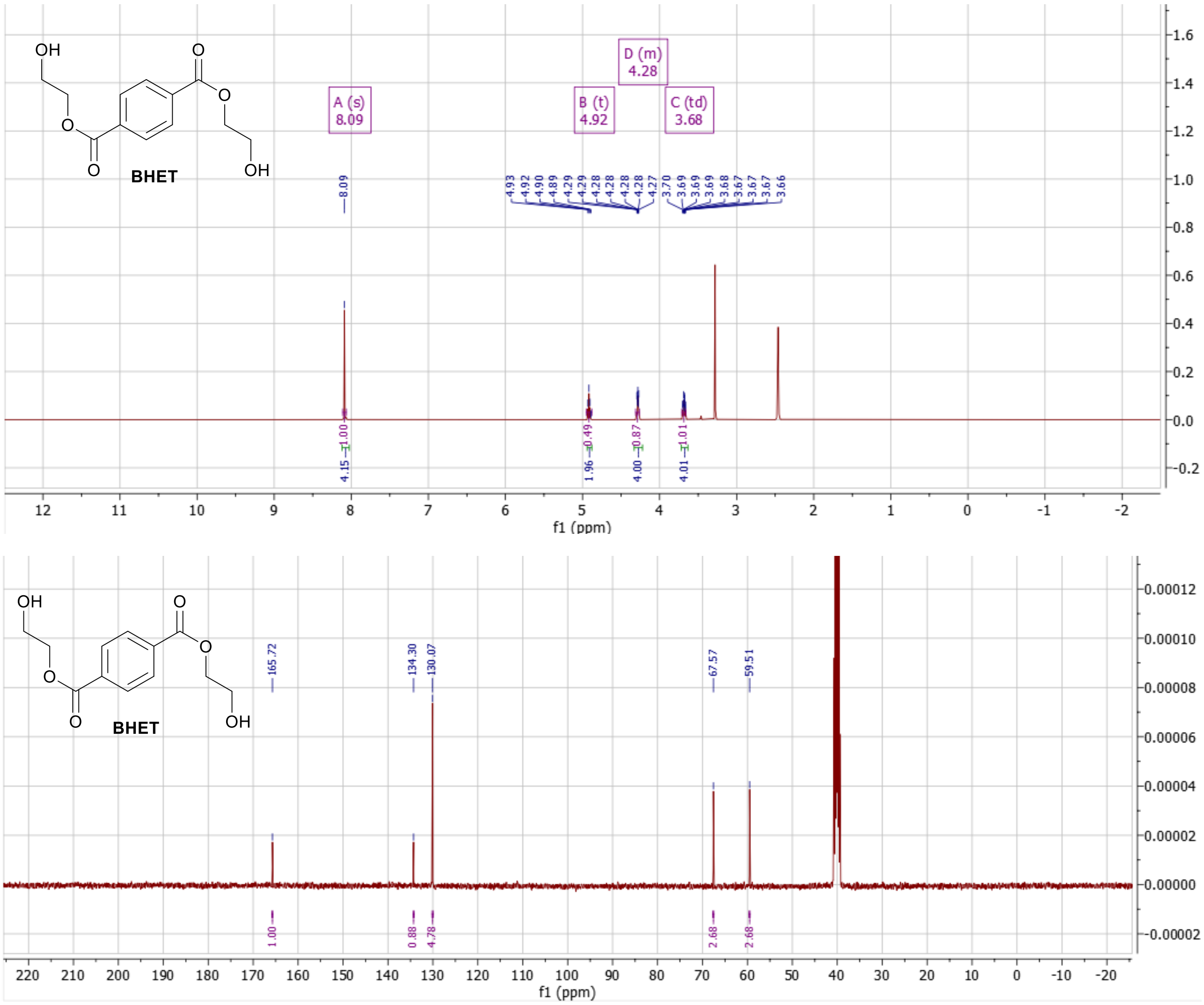

Figure S1. ^{1}H and ^{13}C NMR of Bis hydroxyethyl terephthalate

2-hydroxyethyl terephthalic acid

MW: 210.19 g/mol

White solid, did not melt.

^{1}H NMR (400 MHz, DMSO-d_6) δ 8.08 – 8.01 (m, 4H), 4.27 (dd, J = 5.8, 4.0 Hz, 2H), 3.67 (dd, J = 5.8, 4.0 Hz, 2H).

^{13}C NMR (101 MHz, DMSO-D_6) δ 167.18 (CO), 165.80 (CO), 135.57 (Cq), 133.86 (Cq), 130.07 (2CH), 129.98 (2CH), 67.49 (CH_2), 59.51 (CH_2).

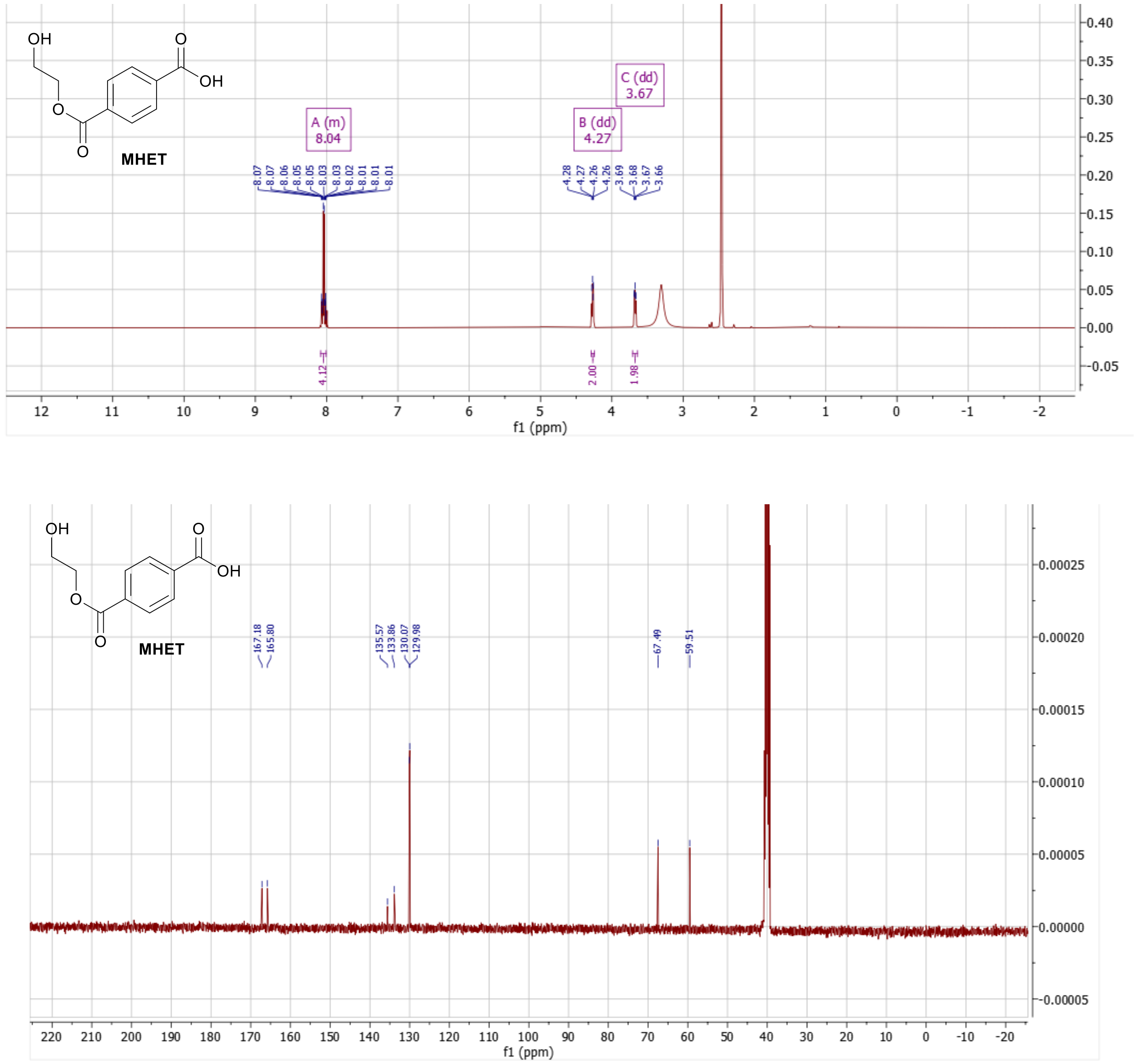

Figure S2. ^{1}H and ^{13}C NMR of 2-hydroxyethyl terephthalic acid

Bis hydroxyethyl furan-2,5-dicarboxylate

MW: 243.93 g/mol

White solid, MP: 102-107°C

^{1}H NMR (400 MHz, DMSO-d_6) δ 7.41 (s, 2H), 4.91 (OH) (t, J = 5.5 Hz, 2H), 4.30 – 4.23 (m, 4H), 3.64 (q, J = 5.3 Hz, 4H).

^{13}C NMR (101 MHz, DMSO-d_6) δ 158.08 (2CO), 146.75 (2Cq), 119.71 (2CH), 67.51 (2CH_2), 59.37 (2CH_2).

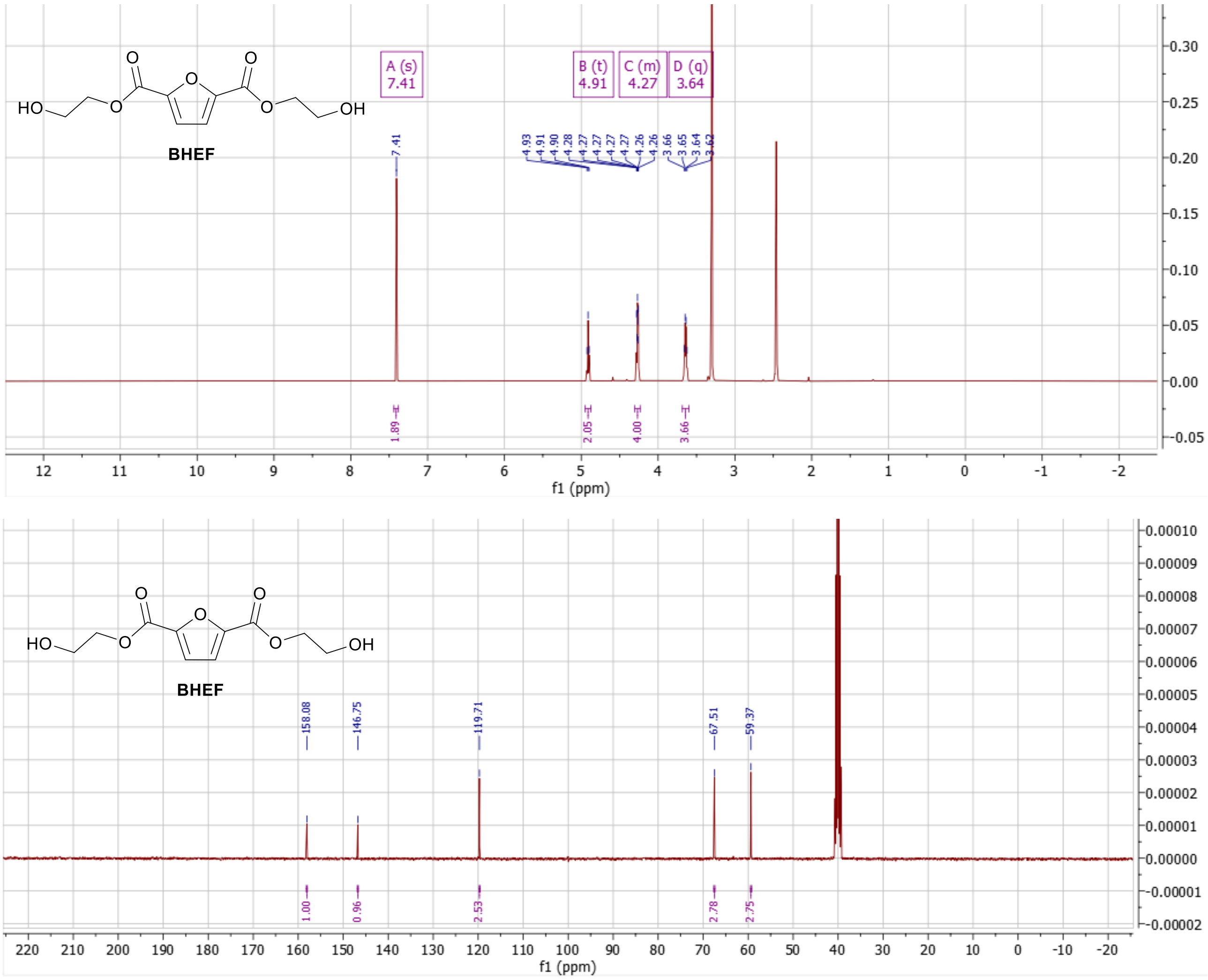

Figure S3. ^{1}H and ^{13}C NMR of Bis hydroxyethyl furan-2,5-dicarboxylate

2-hydroxyethyl furan-5-carboxylic acid

MW: 200.15 g/mol

^{1}H NMR (400 MHz, DMSO-d_6) δ 7.20 (s, 1H), 6.80 (s, 1H), 4.20 (t, J = 4.9 Hz, 2H), 3.62 (t, J = 5.0 Hz, 2H).

^{13}C NMR (101 MHz, DMSO-D_6) δ 158.85 (2CO), 143.31 (2Cq), 119.68 (CH), 113.98 (CH), 66.77 (CH_2), 59.44 (CH_2).

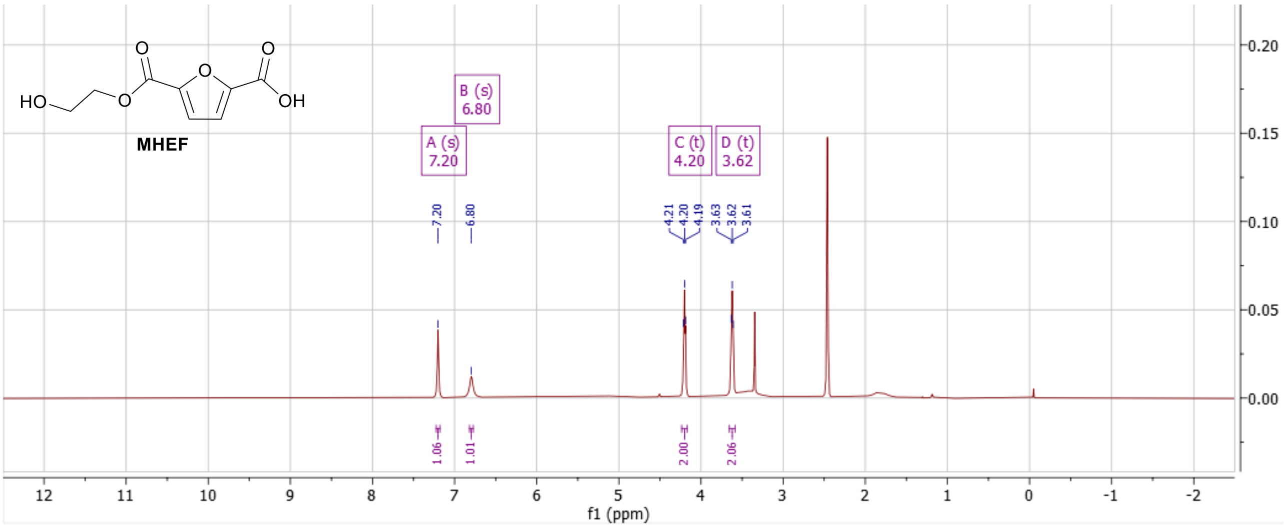

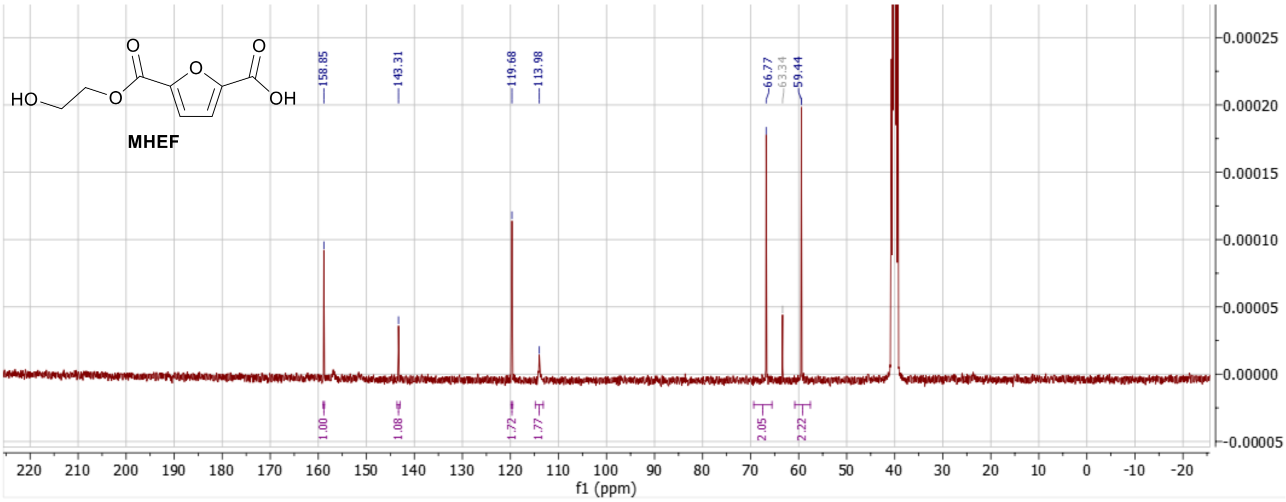

Figure S4. ^{1}H and ^{13}C NMR of Bis hydroxyethyl furan-2,5-dicarboxylate. Peak at 63.34 ppm is residual ethylene glycol (2CH_2).

HPLC-MS analysis

Bis hydroxyethyl terephthalate

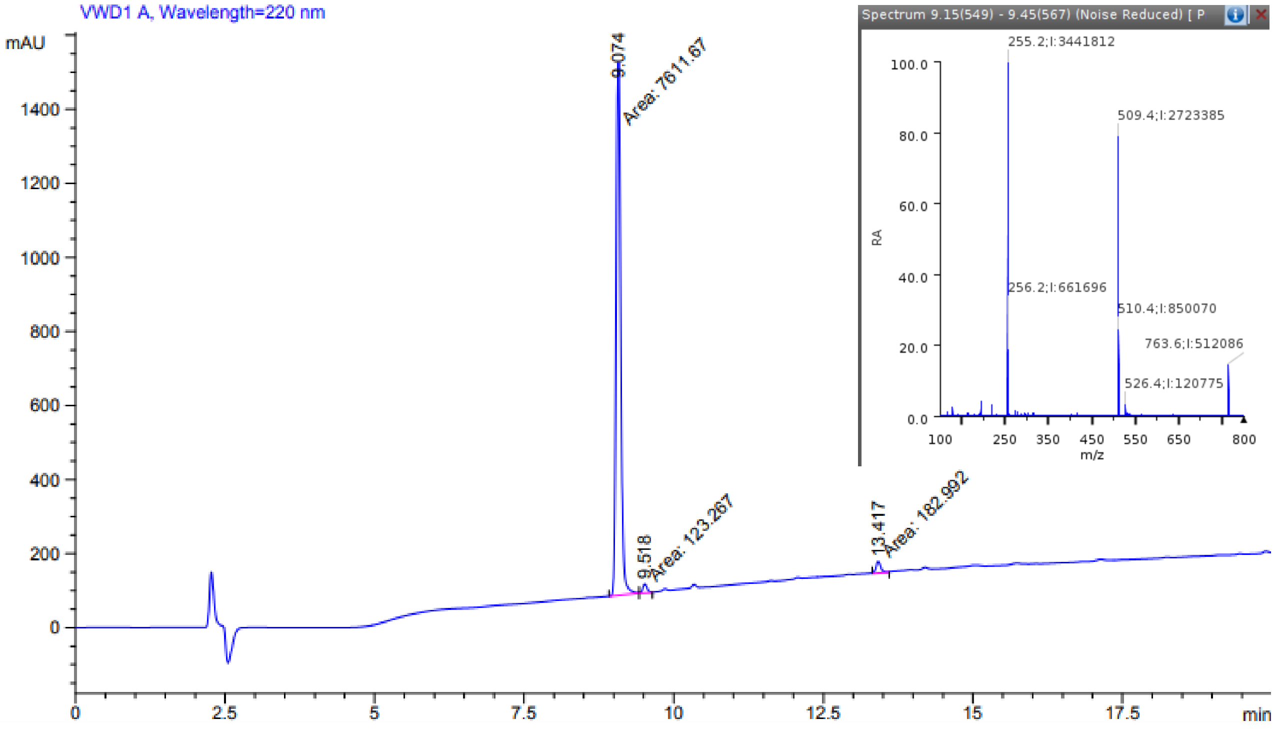

Figure S5. Liquid chromatography and mass spectrometry report of Bis hydroxyethyl terephthalate

Bis hydroxyethyl furan-2,5-dicarboxylate

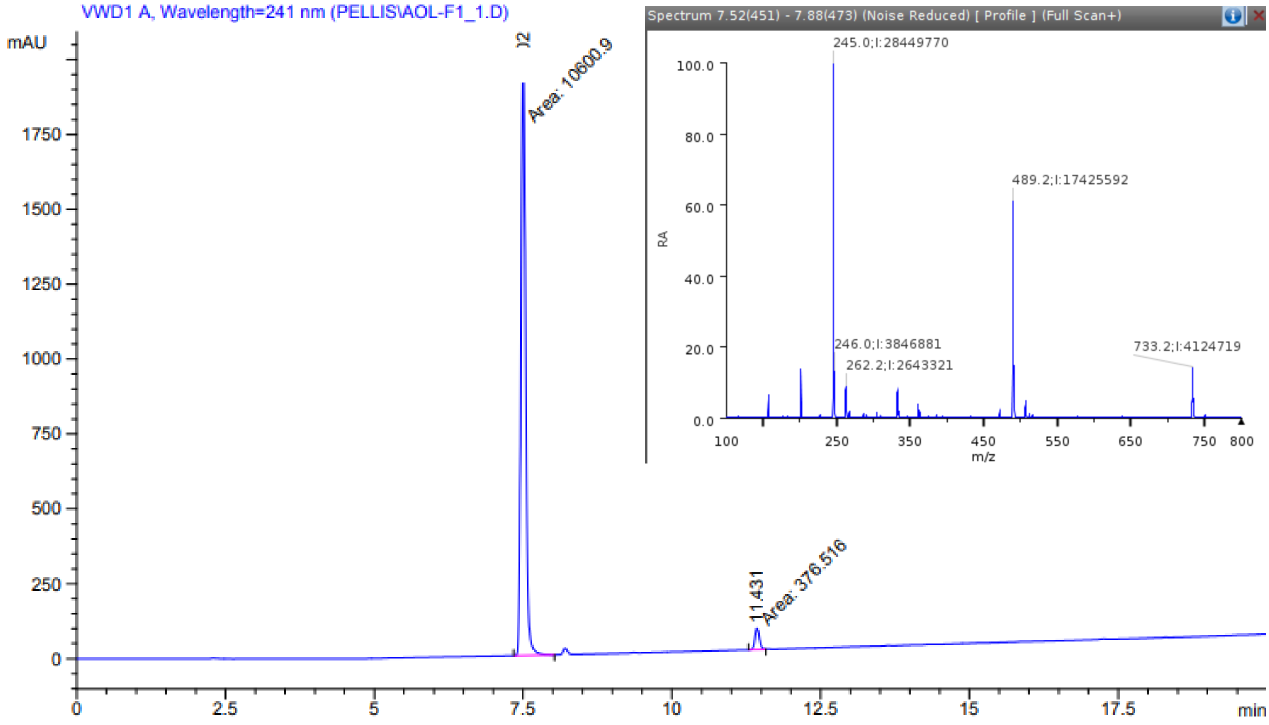

Figure S6. Liquid chromatography and mass spectrometry report of Bis hydroxyethyl furan-2,5-dicarboxylate

2-hydroxyethyl terephthalic acid

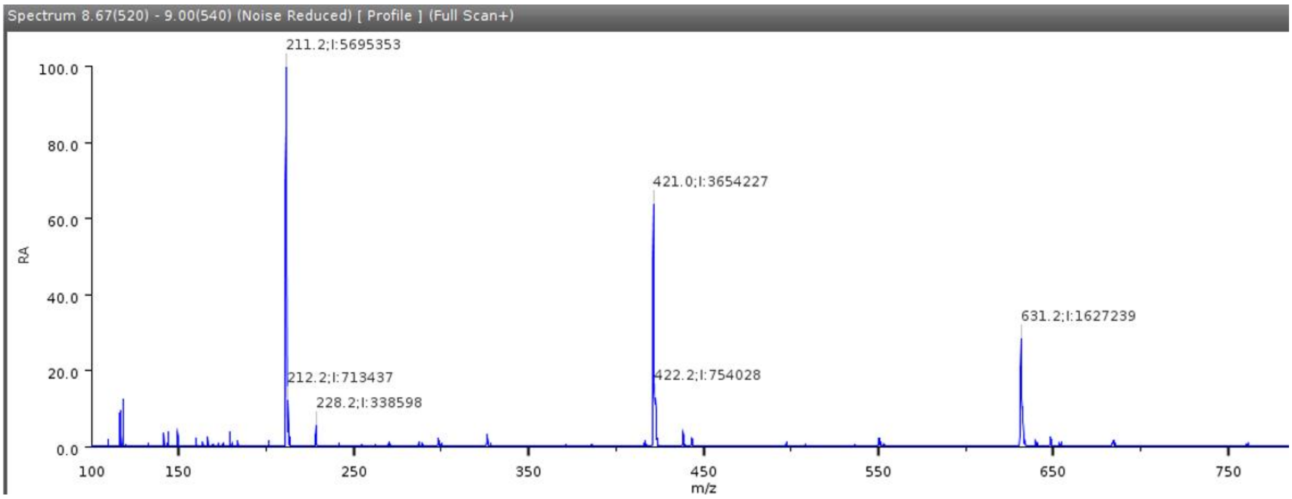

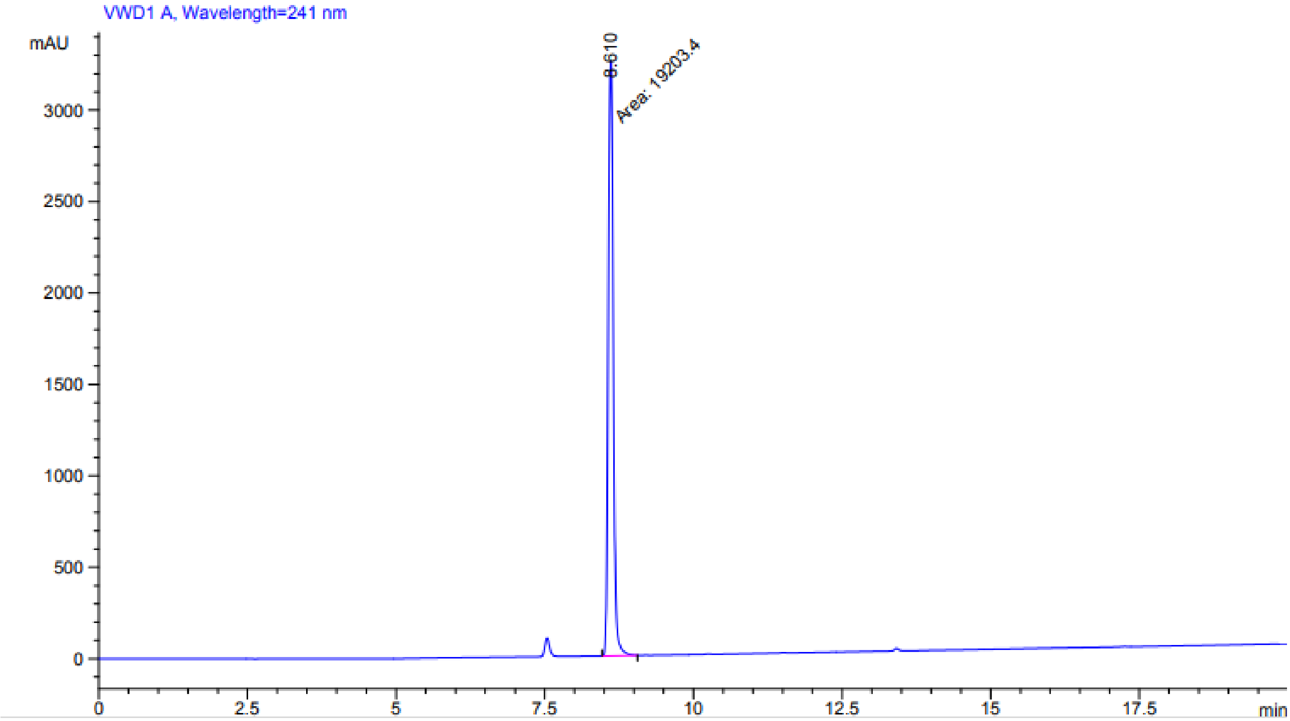

Figure S7. Liquid chromatography and mass spectrometry report of 2-hydroxyethyl terephthalic acid

2-hydroxyethyl furan-5-carboxylic acid

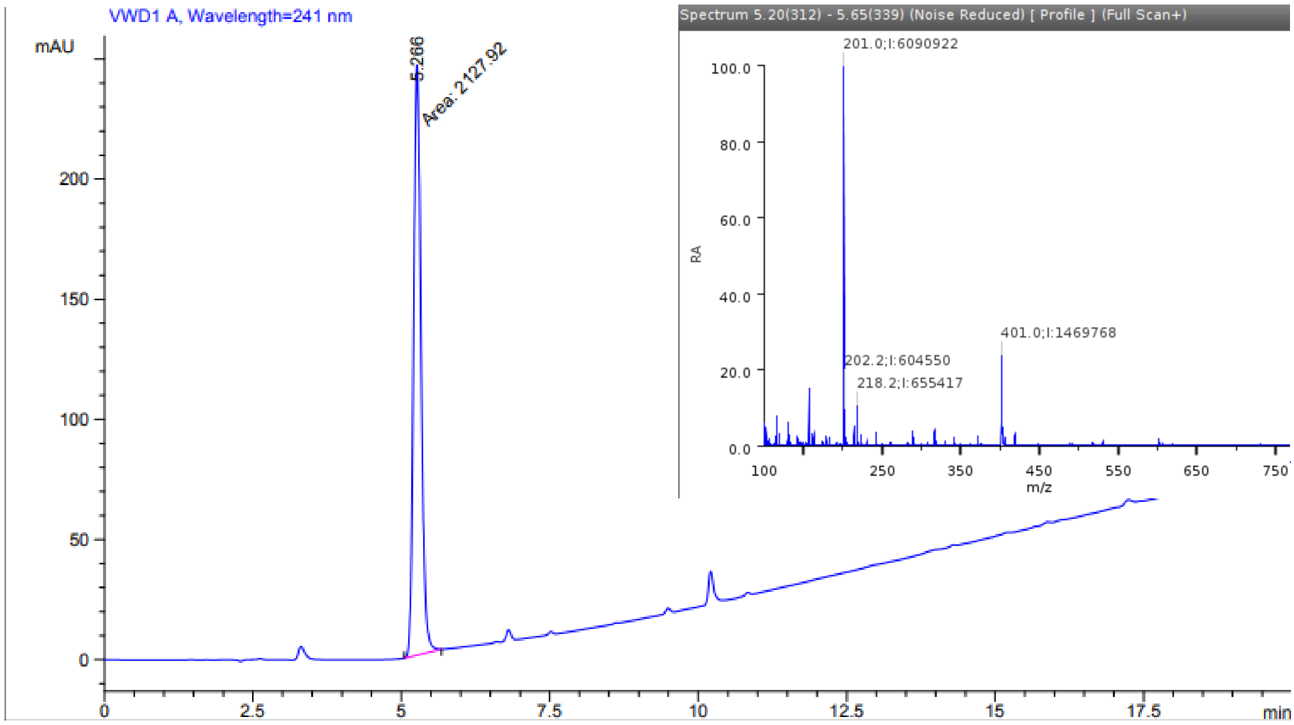

Figure S8. Liquid chromatography and mass spectrometry report of 2-hydroxyethyl furan-5-carboxylic acid

IR report

Bis hydroxyethyl terephthalate

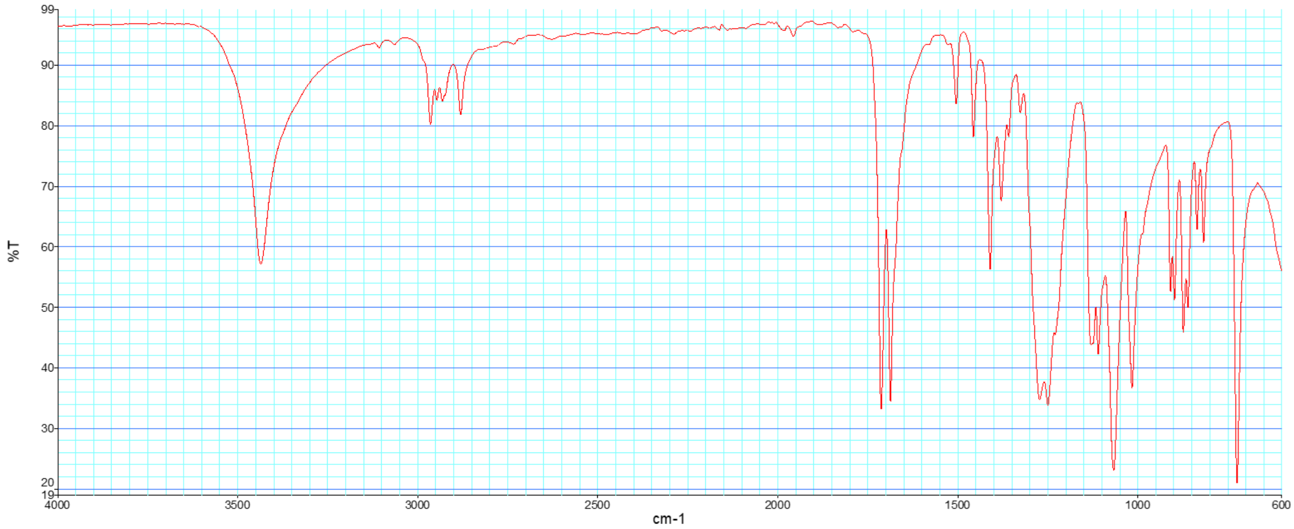

Figure S9. IR spectra of Bis hydroxyethyl terephthalate

Bis hydroxyethyl furan-2,5-dicarboxylate

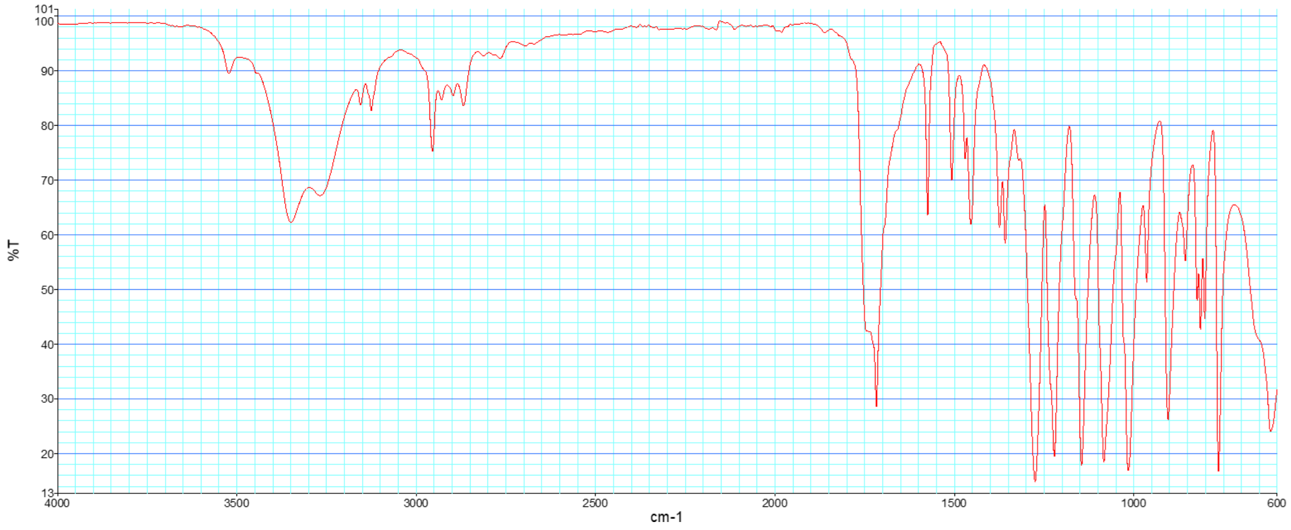

Figure S10. IR spectra of Bis hydroxyethyl furan-2,5-dicarboxylate

2-hydroxyethyl terephthalic acid

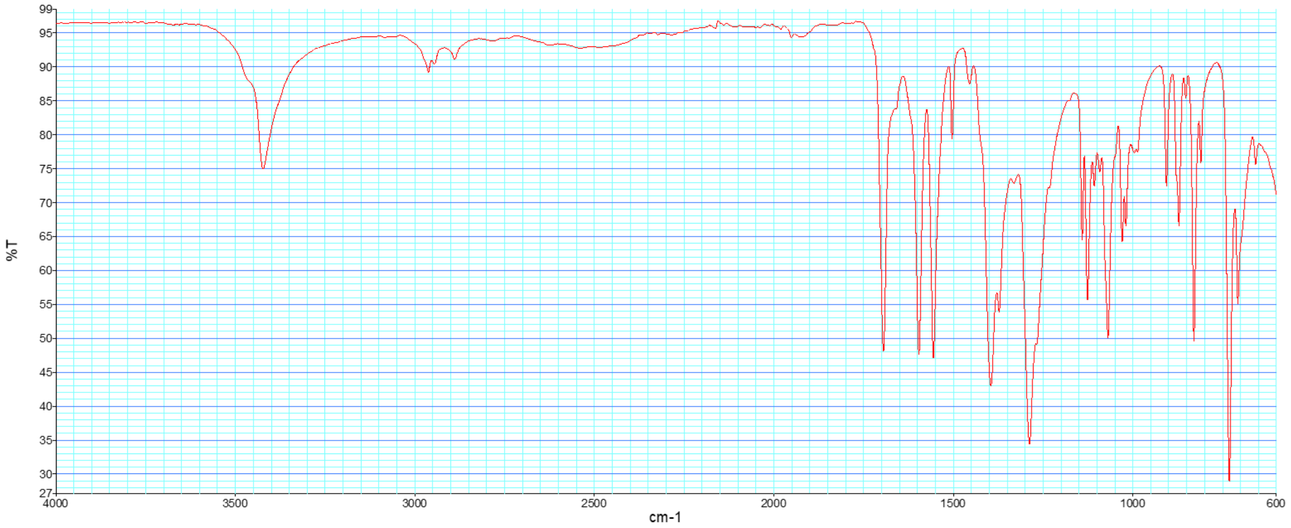

Figure S11. IR spectra of 2-hydroxyethyl terephthalic acid

2-hydroxyethyl furan-5-carboxylic acid

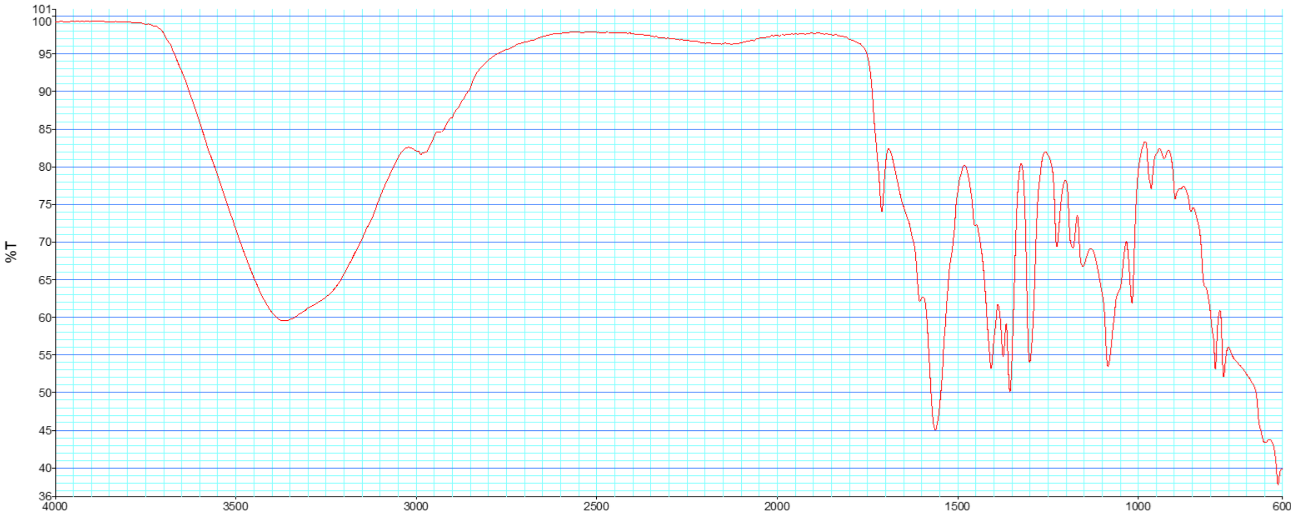

Figure S12. IR spectra of 2-hydroxyethyl furan-5-carboxylic acid
